# Tonsillar metastasis of nonsmall cell lung cancer with G719S mutation in exon 18: A case report

**DOI:** 10.1097/MD.0000000000009003

**Published:** 2017-12-08

**Authors:** Yimin Wu, Zhouyu Zhu, Yongyuan Chen, Ying Chai

**Affiliations:** Department of Thoracic Surgery, the Second Affiliated Hospital, College of Medicine, Zhejiang University, Hangzhou, China.

**Keywords:** G719S mutation, lung cancer, tonsillar metastasis

## Abstract

**Rationale:**

Lung cancer has the highest mortality of all malignant tumors and is becoming the leading cause of death in China. Surgical resection is the best treatment for early non-small-cell lung carcinoma. But postoperative tumor recurrence is very common. Brain, bone and liver are the most common metastatic sites of lung cancer.

**Patient concerns:**

A 59-year-old woman was admitted to our hospital finding a lung nodule in physical examination. No other obvious symptoms were obsessed in this patient. No remarkable abnormality was detected in preoperative laboratory tests and physical examination.

**Diagnoses:**

A ground-glass nodule was detected on the left inferior lobe in the imaging examination. No metastases were detected before the surgery and early-stage lung cancer was supposed.

**Intervention:**

This patient underwent a radical resection of lung cancer successfully and enjoyed a peaceful postoperative rehabilitation.

**Outcomes:**

Although pathological diagnosed confirmed early stage lung adenocarcinoma (T1N0M0). The patient had tumor recurrence 7 months after operation. Gene sequencing confirmed the G719S mutation in exon 18 of the *EGFR* gene and target therapy, chemotherapy and radiotherapy were all given to this patient successively, but they were all unresponsive. The patient died 26 months after surgery.

**Lessons:**

We herein first report G719S mutation in lung adenocarcinoma with tonsillar metastasis. Generally, the tumor responded poorly to treatment and progressed quickly, which didn’t achieve the desired effect. G719S mutant is supposed to be the cause of poor responsive to treatment.

## 1 Introduction

Lung cancer is the leading cause of cancer-related death in China and second most common in women after breast cancer.^[1]^ According to histological type, 2 broad classes are distinguished: nonsmall cell lung carcinoma (NSCLC) and small cell lung carcinoma. Surgical resection is the best method of treatment for early-stage NSCLC. However, tumor recurrence is very common after surgery. Tumor, node, and metastasis (TNM) stage, histology, and mutation status are significant predictors of tumor recurrence and prognosis.^[2]^ Primary lung cancers most commonly metastasize to the brain, bones, liver, and adrenal glands.^[3]^

Tumor recurrence after treatment is becoming a vital point to prolong the overall survival nowadays. Chemotherapy used to be an effective and widely used treatment for patients with tumor recurrence. During the last decade, target therapy and immunotherapy have been approved for the treatment of lung cancer. With an increasing understanding of the pathogenesis, combined modality therapy has been increasingly identified by clinicians.

In this paper, we will give an introduction to a female patient who received a radical surgery and was pathology diagnosed as lung adenocarcinoma. However, 8 months after surgery, a tonsillar metastasis was detected as a mass arising from the right tonsil. Postoperative pathology confirmed tonsillar metastasis of lung cancer. Gene sequencing confirmed the G719S mutation in exon 18 of the epidermal growth factor receptor (*EGFR*) gene and the patient received target therapy with icotinib. Nevertheless, 14 months after surgery, this patient presented with multiple brain metastases. Radiotherapy and chemotherapy were given to this patient then. But they were not very responsive either. Finally, the patient died of respiratory failure 26 months after surgery.

## 2 Case presentation

**Figure 1:**
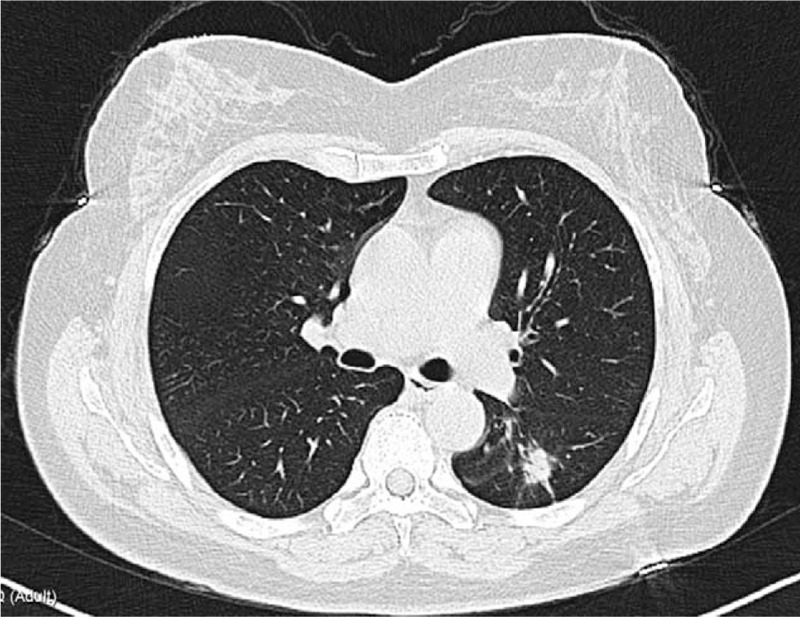
Chest computed tomography scan of this patient detected a ground-glass nodule (2 × 1.5 × 1.5 cm) on the left inferior lobe.

A 59-year-old woman was admitted to our hospital for finding a lung nodule in physical examination. In the laboratory tests and physical examination, no remarkable abnormality was detected and the patient's blood pressure, serum potassium, blood glucose level, and glucose tolerance were all in normal range. No remarkable medical, family, and psychosocial history was observed. The computer tomography (CT) scan identified a ground-glass nodule (2 × 1.5 × 1.5 cm) on the left inferior lobe (Fig. 1). No enlarged lymph nodes or distant metastasis were detected on contrast-enhanced CT. Brain magnetic resonance imaging (MRI) and bone scintigraphy did not detect any other metastases.

**Figure 2:**
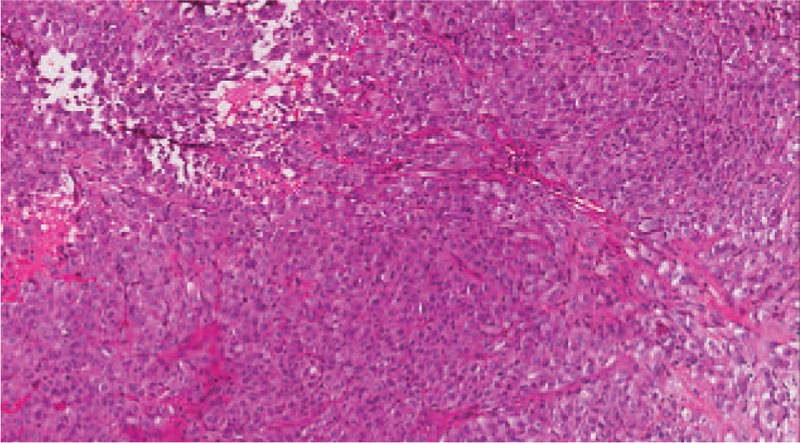
Histopathological examination of the surgical specimen revealed abnormal differentiated glandular structure with staining positive for mucin (hematoxylin and eosin staining, original magnification ×10).

Being primary diagnosed with early-stage lung cancer, the patient received a wedge resection and the intraoperative rapid frozen section confirmed lymphoma, and then the tumor was completely resected through lobectomy and systematic mediastinal lymphadenectomy. Grossly, the 2 tumor foci measured 1.5 × 2 cm in size. Histologically, the tumor consisted of abnormal differentiated glandular structure with staining positive for mucin (Fig. 2). Immunohistochemically, CD7, TTF-1, NapsinA were positive and Ki-67 labeling index was 30%. On the basis of these features, the diagnosis of lung adenocarcinoma was made (T1N0M0).

**Figure 3:**
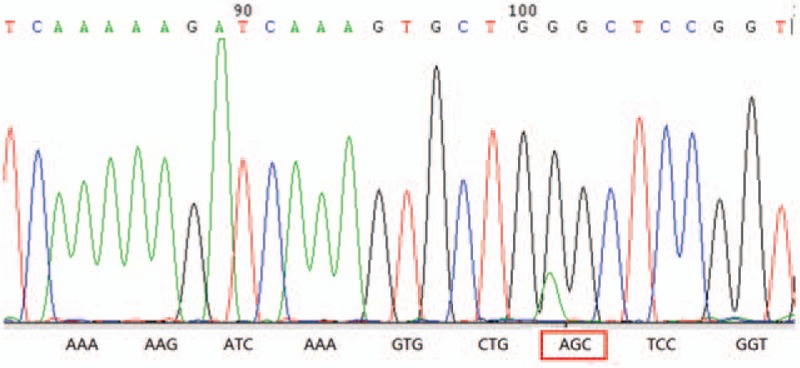
G719S mutation in exon 18 of the *EGFR* gene detected by direct Sanger sequencing of biopsied samples from the resected tonsil. It causes a flame shifting change with glycine (The codon change from GGC to AGC).

However, 8 months after surgery, this patient coughed up a bloody mass. She received laryngealendoscopy examination and it detected a mass arising from the right tonsil. An incisional biopsy of tonsillar lesion suggested metastasis of the carcinoma from the palatine tonsils to the cervical lymph. The patient underwent a tonsillectomy and the pathology confirmed the tonsillar metastasis of lung cancer. Gene sequencing confirmed the G719S mutation in exon 18 of the *EGFR* gene and the patient received target therapy with icotinib (Fig. 3). Whereas, multiple brain metastases were detected in brain MRI 14 months after surgery and then the patient received radiotherapy (DT 2000cGy) for 10 times. After that, chemotherapy (pemetrexed and platinum) for 4 cycles, bevacizumab and chemotherapy was also given to this patient successively. But they all did not work well. Finally, the patient died of respiratory failure 26 months after surgery.

## 3 Discussion

Tumor recurrence after surgery of lung cancer is quite common. Therefore, clinicians have focused more recently on additional treatments after tumor recurrence. Treatments for advanced or recurrent NSCLC included cytotoxinic chemotherapy with platinum-based agents, EGFR-tyrosine kinase inhibitors (TKIs) for EGFR mutant patients, crizotinib for *ALK* gene rearrangement, and so on.^[4]^ Individual comprehensive therapy is becoming a mainstream in the treatment of advanced NSCLC nowadays.

In our case, pathological stage was just T_1_N_0_M_0_. Immunohistochemically, Ki-67 labeling index was only 30%. Generally, this patient should enjoy a good prognosis.^[2,5]^ However, tonsillar metastasis occurred just 8 months after surgery, which is a very rare metastatic site of lung cancer. There are no systematic data about the prognosis of lung cancer patients with tonsillar metastasis. Thereafter, the patient received target therapy, radiotherapy, and chemotherapy successively. But the duration of therapy is unsatisfactory. The tumor recurred soon after surgery and progress very fast, what is beyond our expectation.

Except TNM staging and pathological type, we found that gene sequencing confirmed G719S mutation in this patient. G719S mutation located in exon 18 of EGFR and can cause a flame shifting change with glycine and it turns to be serine and it accounts for 2% of all EGFR mutations in lung cancer.^[6]^ In a preclinical study, it shows the oncogenic potential and EFGR-TKIs sensitivity.^[7]^

However, target therapy with EGFR-TKIs is not very effective in our case. Moreover, radiotherapy and chemotherapy did not work well either. We suppose that G719S mutant may be the cause of poor responsive to treatment. The overall progression-free survival (PFS) of patients with G719S mutant is just 4 months, which is lower than median PFS of EGFR-mutated patients.^[8,9]^ One possible mechanism to explain this is the EGFR-TKIs binds looser to EGFR G719S mutation than to L858R mutation, the most mutation in NSCLC.^[6,7]^ Some clinical studies also indicate that G719S mutation is somewhat more resistant to gefitinib.^[8]^

Although current study indicates G719S mutant may be a poor prognostic factor of lung adenocarcinoma, specific treatment for such patients is not taken seriously yet. Some literatures suggest that afatinib and neratinib may be more sensitive to EGFR G719S mutation.^[10,11]^ But it warrants further validation.

## Acknowledgment

The authors declare that they have no institution to acknowledge.

## References

[1] Chen WR Zheng PD Baade S ’Cancer statistics in China, 2015 CA Cancer J Clin 2016; 66:115–132. 10.3322/caac.21338 26808342

[2] Alexander M Wolfe R Ball D Lung cancer prognostic index: a risk score to predict overall survival after the diagnosis of non-small-cell lung cancer. Br J Cancer 2017;117:744–51. 28728168 10.1038/bjc.2017.232 PMC5572183

[3] Lu C Onn A Vaporciyan AA “Chapter 78: Cancer of the Lung”. Holland-Frei Cancer Medicine (8th). People's Medical Publishing House, USA. 2010. ISBN 9781607950141.

[4] Ettinger DS Wood DE Aisner DL Non-small cell lung cancer, version 5.2017, NCCN Clinical Practice Guidelines in Oncology. J Natl Compr Canc Netw 2017;15:504–35. 28404761 10.6004/jnccn.2017.0050

[5] Kadota K Yeh YC Sima CS The cribriform pattern identifies a subset of acinar predominant tumors with poor prognosis in patients with stage I lung adenocarcinoma: a conceptual proposal to classify cribriform predominant tumors as a distinct histologic subtype. Mod Pathol 2014;27:690–700. 24186133 10.1038/modpathol.2013.188 PMC4374572

[6] Kris MG Johnson BE Berry LD Using multiplexed assays of oncogenic drivers in lung cancers to select targeted drugs. JAMA 2014;311:1998–2006. 24846037 10.1001/jama.2014.3741 PMC4163053

[7] Greulich H Chen TH Feng W Oncogenic transformation by inhibitor-sensitive and -resistant EGFR mutants. PLoS Med 2005;2:e313. 16187797 10.1371/journal.pmed.0020313 PMC1240052

[8] Otsuka T Mori M Yano Y Effectiveness of tyrosine kinase inhibitors in Japanese patients with non-small cell lung cancer harboring minor epidermal growth factor receptor mutations: results from a multicenter retrospective study (HANSHIN Oncology Group 0212). Anticancer Res 2015;35:3885–91. 26124334

[9] Mitsudomi T Morita S Yatabe Y Gefitinib versus cisplatin plus docetaxel in patients with non-small-cell lung cancer harbouring mutations of the epidermal growth factor receptor (WJTOG3405): an open label, randomised phase 3 trial. Lancet Oncol 2009;11:121–8. 20022809 10.1016/S1470-2045(09)70364-X

[10] Sequist LV Besse B Lynch TJ Neratinib, an irreversible pan-ErbB receptor tyrosine kinase inhibitor: results of a phase II trial in patients with advanced non-small-cell lung cancer. J Clin Oncol 2010;28:3076–83. 20479403 10.1200/JCO.2009.27.9414

[11] Yang JC Sequist LV Geater SL Clinical activity of afatinib in patients with advanced non-small-cell lung cancer harbouring uncommon EGFR mutations: a combined post-hoc analysis of LUX-Lung 2, LUX-Lung 3, and LUX-Lung 6. Lancet Oncol 2015;16:830–8. 26051236 10.1016/S1470-2045(15)00026-1

